# Opinion: Yes

**DOI:** 10.1590/S1677-5538.IBJU.2015.06.03

**Published:** 2015

**Authors:** Marcelo Vieira

**Affiliations:** 1 Membro Titular da Sociedade Brasileira de Urologia

**Keywords:** Vasovasostomy, Sterilization Reversal, Infertility, Azoospermia, Fertility

Decision on which is the best treatment for a problem that has more than one way to be dealt with goes much further from exclusively looking to the final results. Decision must include which are the final results you are looking for, how many variables are present in both options, money expenditure to reach the result and finally, how much resources are available for it.

The beginning of 90's brought consolidation of vasectomy reversal as the treatment for man who had a vasectomy and want to become a father again. With the paper publishing with published paper from Belker and cols. that established time from vasectomy as a main variable for patency rate and pregnancy after surgery (1).

In 1992 the first pregnancy obtained from an injection of a single sperm into a oocyte was published by Palerno et al. (Intracitoplasmic Sperm Injection – ICSI) (2) and two years later Shrivrastav and cols. published the technique for sperm retrieval from epididymis by a percutaneous puncture (Percutaenous Epididymal Sperm Aspiration – PESA) (3), introducing a second option for man with vasectomy to become a father and new variables that may alter the results. ICSI/PESA introduced variables that come from the female partner, mainly age, and laboratory variables as the number of metaphase oocytes manipulated, fertilization rate and embryo classification (4–7).

The final objective of infertile couples seeking for treatment is having a healthy baby and not only getting pregnant, so, papers objecting the comparison between vasectomy reversal and ICSI with sperm retrieval (SR) must also look for live birth rate. Silber and cols., in 1995 published the results of 72 cycles of Microsurgical Sperm Aspiration (MESA) and ICSI showing a 42% ongoing pregnancy rate. Since then the literature shows pregnancy rates ranging from 28 to 42% and live birth from 27 to 32% (7–13). Data from Society for Assisted Reproductive Technology (SART) shows that live birth rate for male factor was 34.5 % in 2003 and raised to 37.3% in 2008 and the use of ICSI for male factor raised from 84% to 87%, but those numbers are for all male factors (14).

Lee and cols. published a literature review comparing vasectomy reversal and sperm retrieval (SR) /ICSI and showed a data compilation of reversal results with patency rate range from 60 to 90% and pregnancy rate range from 26 to 84% with median patency rate of 86% and pregnancy rate of 56%. Excluding the best and worst results from the analysis the median patency rate was 81% and pregnancy rate 44% (15).

The pregnancy and live birth rates are very similar and the analysis of prognostic variables may help taking a decision towards the most suitable. Belker and cols. showed that time from vasectomy is the prognostic factor for patency rate (1). Four papers studied time from vasectomy as a prognostic factor for pregnancy and live birth after SR/ICSI for man with vasectomy (9–12). Borges et al. and Abdelmassih results showed that time since vasectomy was a prognostic factor for pregnancy and Bromage et al. and Nicopoullos et al. found just the opposite, but all agreed that female partner age was an important prognostic factor. Friedler et al. studied the prognostic factors for ICSI with sperm retrieval in obstructive azoospermia and concluded that the female age and number of metaphase oocytes manipulated were the best indicators for pregnancy and live birth. So female age is important and correlates with pregnancy and live birth for SR/ICSI. Butwhat about the role of female age as a prognostic factor for pregnancy and live birth after vasectomy reversal?

Hsieh et al., using a decision analysis model to choose between SR/ICSI and vasectomy reversal concluded that female age was more important than time since vasectomy for pregnancy rate and live birth after a vasectomy reversal and the correlation is also true for SR/ICSI, so younger female will perform better in both scenarios leaving us without one best option (16).

The last two aspects for decision making is the cost-effectiveness, a concept of how much resources do we spend to achieve a certain result. In our case live birth is one and the other one is how much money the couple intends to spend to have a child called “willing to pay” (WTP) (16, 17). Those two concepts are addressed in cost-effectiveness studies which are very few in the literature. Search on PUBMED ((“Vasovasostomy/economics”[Mesh] or “Vasovasostomy/statistics and numerical data”[Mesh])) AND “Cost-Benefit Analysis”[Mesh] resulted in 12 papers. Three were excluded from the analysis: one in German, one animal model study and one which analyzed vasectomy as a reversible method. After reading the other 10, we excluded two more for not address the subject.

Garceau et al. in a systematic review of assisted reproductive technology, elected 59 papers addressing economics evaluations, cost studies and economic benefits among 2.547 papers searched on the literature. The authors extracted the economic data, converted to UK pounds and adjusted inflation with the objective of analyzes cost for assisted reproductive technology, including cost per delivery after vasectomy reversal and SR/ICSI. The authors found a median cost per delivery of £ 42.163,50 for SR/ICSI and £ 16.134,00 for a vasectomy reversal. Despite the small sample from one of the papers, 25% lost follow up from another and the median (50-80%) of quality requirements from a great number of papers, they concluded on behalf of the superiority of vasectomy reversal (17). SR/ ICSI would be more cost-effective if the pregnancy rate was higher or the couples would have more money to spend (WTP). Hsieh and cols. with a simulation decision model using the available data on the literature and working with diversified scenarios of female age and vasectomy reversal patency rates, concluded that IVF is more effective for pregnancy rate over 60% and vasectomy reversal has an advantage until US$ 65.000 of WTP (16). The most recent published reviews also confirmed vasectomy reversal as a first line treatment for vasectomy (14, 18).

Some facts must be discussed as advantage and disadvantage for both treatments. Lee and cols. as Roob & Sandlow and Shridharani & Sandlow considered direct and indirect costs of IFV that inflates IVF cost explaining the advantage for vasovasostomy. The need for contraception after a successful vasectomy reversal may lead the couples to SR/ICSI but might also be counter pointed by the higher chance for multiple births after IFV. Specialized centers have better results either for IVF and reversal. Limitations of cost-effectiveness studies due to different fares and regional coverage for ART treatment in US may bias the option for one or other treatment. Studies concentrated on US and developed countries may not be generalized for other countries. We do not have cost-effectiveness studies in Brazil analyzing SR/ICSI and vasectomy reversal (14–16, 18).

Our center has both options for man with vasectomy, and the decision of which treatment will be done, is ultimately taken by the couple after knowing the results and analyzing costs. From 1994 until 2012 four hundred and fifty SR/ICSI cycles were done for man with vasectomy to treat 332 couples, 318 for the first time. The pregnancy rate per cycle was 31%(141/450) and the live birth per cycle was 22% (10/450). Cumulative pregnancy rate and live birth rate was 42% (141/332) and 30 %(102/332) respectively. Couples were submitted up to seven cycles and all the live births occurred until the fourth cycle, just one pregnancy after four cycles and none live birth after four cycles done (19). Since 2008 we did 166 vasectomy reversals, 147 data available for semen analysis results with 83% patency rate (123/147). A hundred and two patients could be contacted by phone for late follow up and amongst that, 40 healthy babies were born, live birth rate 39% (40/102).

All these factors can lead us to conclude to either side but common sense and until now, published results still reaffirm reversal as a primary treatment for vasectomy.
